# Telomere-to-telomere *Schizosaccharomyces japonicus* genome assembly reveals hitherto unknown genome features

**DOI:** 10.1002/yea.3912

**Published:** 2024-03-07

**Authors:** Graham J. Etherington, Pei-Shang Wu, Snezhana Oliferenko, Frank Uhlmann, Conrad A. Nieduszynski

**Affiliations:** 1 The Earlham Institute, Norwich, UK; 2 The Francis Crick Institute, London, UK; 3 Randall Centre for Cell and Molecular Biophysics, School of Basic and Medical Biosciences, King’s College London, London, UK; 4 Cell Biology Centre, Institute of Innovative Research, Tokyo Institute of Technology, Kanagawa, Japan; 5 School of Biological Sciences, University of East Anglia, Norwich, UK

**Keywords:** centromere, fission yeast, rDNA, *Schizosaccharomyces japonicus*, telomere, tRNA

## Abstract

*Schizosaccharomyces japonicus* belongs to the single-genus class Schizosaccharomycetes, otherwise known as “fission yeasts.” As part of a composite model system with its widely studied *S. pombe* sister species, *S. japonicus* has provided critical insights into the workings and the evolution of cell biological mechanisms. Furthermore, its divergent biology makes *S. japonicus* a valuable model organism in its own right. However, the currently available genome assembly contains gaps and has been unable to resolve centromeres and other repeat-rich chromosomal regions. Here we present a telomere-to-telomere long-read genome assembly of the *S. japonicus* genome. This includes the three megabase-length chromosomes, with centromeres hundreds of kilobases long, rich in 5S ribosomal RNA genes, transfer RNA genes, long terminal repeats, and short repeats. We identify a gene-sparse region on chromosome 2 that resembles a 331 kb centromeric duplication. We revise the genome size of *S. japonicus* to at least 16.6 Mb and possibly up to 18.12 Mb, at least 30% larger than previous estimates. Our whole genome assembly will support the growing *S. japonicus* research community and facilitate research in new directions, including centromere and DNA repeat evolution, and yeast comparative genomics.

## 1 Introduction

Fission yeasts are a single-genus group of yeasts within the Taphrinomycotina subdivision of *Ascomycota* fungi, which divide equatorially into two daughter cells of equal size (Taylor & Berbee, 2006). The *Schizosaccharomyces* genus contains a well-established model organism *S. pombe*, together with *S. octosporus,S. cryophilus, S. osmophilus, S. lindneri, S. versatilis*, and an emerging model system *S. japonicus*. All known species of fission yeast have now been sequenced (Brysch-Herzberg et al., 2023; Jia et al., 2023; Rhind et al., 2011; Wood et al., 2002; Yukawa & Maki, 1931).

*S. pombe* is a long-established model organism utilised across a wide range of cellular and molecular biology research (Rutherford et al., 2022; Wood et al., 2002, 2012). Despite being closely related and relying on many conserved genes, several major differences with *S. japonicus* has made this pair of sister species emerge as a powerful evolutionary cell biology system (Alam et al., 2023; Gu et al., 2015; Makarova et al., 2016, 2020; Yam et al., 2011). Furthermore, *S. japonicus* has become a standalone model organism for study of processes not present or tractable in other model yeasts, such as mitotic nuclear envelope breakdown and reassembly, cellular geometry scaling and the RNA interference pathway controlling both post-transcriptional gene silencing and heterochromatin formation (Aoki et al., 2011; Chapman et al., 2022; Furuya & Niki, 2010, 2012; Gomez-Gil et al., 2019; Gu & Oliferenko, 2019; Kinnaer et al., 2019; Klar, 2013; Lee et al., 2020; Nozaki et al., 2018; Papp et al., 2021; Pieper et al., 2020; Rutherford et al., 2022; Wang et al., 2021; Yam et al., 2013).

A chromosome-level assembly of *S. japonicus* totalling 11.7 Mb has long been available (Rhind et al., 2011) and has recently been utilised to create a valuable online database and research tool for this species—JaponicusDB (Rutherford et al., 2022). The assembly of the three *S. japonicus* chromosomes was inferred from genetic linkage within the seven largest supercontigs in the SJ1 assembly, which were then joined with four stretches of N’s. Three of these joins are centromeres, which were not resolved in the assembly and were substituted by stretches of N’s, 120 kb in length. The final gap, on chromosome 2 between supercontigs 7 and 5, was the last region to be assembled (Rhind et al., 2011). This join lies roughly 400 kb downstream from the centromere. The sequence composition of a 12 kb region in this area contains four further stretches of N’s between 100 bps and 1680 bps in length.

The availability of low-cost long-read sequencing combined with advances in genome assembly algorithms has allowed for chromosome-scale telomere-to-telomere sequencing to become achievable even over complex genomes (Belser et al., 2021; Nurk et al., 2022; Tong et al., 2019). Here we utilise Oxford Nanopore (ONT) long-read sequencing to assemble a telomere-to-telomere reference sequence of *S. japonicus* and compare it to the current version of the reference genome (GCF_000149845.2_SJ5).

## 2 Materials and Methods

### 2.1 Cell culture and high molecular weight DNA extraction

High molecular weight genomic DNA was isolated from a mating type h-isolate of *S. japonicus* strain NIG2028 (Furuya & Niki, 2009), using the Nanobind CBB DNA extraction kit (PacBio), according to the manufacturer’s instructions with modifications. Briefly, *S. japonicus* was cultured in YE media supplemented with 3% glucose, adenine hydrochloride (100 mg/L), histidine (75 mg/L), leucine (75 mg/L), and uracil (75 mg/L) at 30°C. Forty-millilitre culture of exponentially growing cells at OD_600_ = 0.5 were harvested, and washed twice with 0.05 M EDTA pH 8.0 and once with CSE buffer (50 mM citrate-phosphate pH 5.6, 40 mM EDTA and 1.2 M sorbitol). The cells were then treated with zymolyase 100 T (0.6 mg/ml in CSE buffer) at 37°C for 50 min. The subsequent cell lysis and DNA extraction were performed as described in the manufacturer’s protocol for hypocrealean fungi, except that CLE3 buffer was replaced with an optimised Proteinase K buffer (10 mM Tris-HCl pH 9.5, 0.5 M EDTA, 125 mM Na_2_SO_3_, and 1% lauryl sarcosine), while Buffer SB was replaced with 10 mM Tris-HCl, pH 9.5. The size distribution of the extracted genomic DNA was confirmed by pulsed-field gel electrophoresis.

### 2.2 DNA sequencing

DNA concentration and size were assessed using the double-stranded high-sensitivity assay on a Qubit fluorometer and a TapeStation system using a Genomic DNA ScreenTape, respectively. Genomic DNA was sequenced using the GridION nanopore sequencer (Oxford Nanopore Technologies). One sequencing library was generated using a ligation kit (SQK-LSK112) and sequenced on one MinION R10.4 flow cell (FLO-MIN112), following the manufacturer’s guidelines. This generated 3.38 Gb of QC-passed ONT long read data (from 498,295 reads), with an estimated N50 read length of 27.31 kb. 646 reads were greater than 100 kb in length, nine reads were greater than 200 kb, and the maximum read length was 284 kb.

### 2.3 Additional genomic datasets

We used four libraries of publicly available *S. japonicus* Illumina short-read data for sequence polishing (BioProject accession PRJNA770288). This data set was from a genome resequencing experiment of two dcr1 deletion survivor strains that have acquired spontaneous mutations and have gained increased copies of retro-transposons, although we considered the benefit of using this data set for polishing far outweighed the negatives of not polishing. We also downloaded the current publicly available genome assemblies and annotations for *S. japonicus* from NCBI (GCF_000149845.2_SJ5), and JaponicusDB (Rutherford et al., 2022). Although originating from the same initial reference sequence, the two different data sources contain complementary data. The NCBI annotation contains information such as gene biotypes and products, whilst the three JaponicusDB SJ5 chromosome-level supercontigs (5.1, 5.2, and 5.3) have been renamed as chromosomes I, II, and III, respectively, along with the mitochondrial genome.

### 2.4 Genome assembly

We used minimap2 (Li, 2018) to align ONT reads to the *S. japonicus* reference assembly and then used Alvis (Martin & Leggett, 2021) to identify chimeric reads. Alvis identified 4982 reads that appeared to be chimeric, which were removed from the data set. Then, we used Canu (Koren et al., 2017) to correct and trim the ONT reads and then generated three de novo assemblies; one using all the data and one each using reads >50 kb and >60 kb. We then assessed the three assemblies on their completeness and contiguity, expecting the ‘best’ assembly to be that which resolved each of the three *S. japonicus* chromosomes with the fewest contigs as well as assembling the mitochondrial genome. We removed contigs from the assembly that Canu suggested were graph bubbles (“suggestBubble = yes” in the Canu fasta header) and trimmed contigs that were suggested to be circular (“suggestCircular = yes” in the Canu fasta header). Further, we performed a self-versus-self blastn of the mitochondrial genome and trimmed any redundant overlapping sequence (Altschul et al., 1990). Finally, we polished the final assembly with the four libraries of Illumina data using three rounds of Pilon (Walker et al., 2014).

Next, we used the nucmer tool in the MUMmer tool suite (Delcher et al., 2003) to align contigs in the assembly to that of the *S. japonicus* genome from JaponicusDB to identify syntenic chromosomal regions between the two assemblies, visualising the output in Dot (Nattestad, 2020).

We carried out quality control steps to assess the final assembly. As well as calculating contig-specific GC content and contiguity statistics, we also used BUSCO (v5.3.2) (Manni et al., 2021) to identify the number of *Ascomycota*-specific single-copy orthologs and KAT (Mapleson et al., 2016) to compare the distribution of k-mers in the high-accuracy Illumina reads (from BioProject accession PRJNA770288) to that of the assembly, using a mer length of 31. To compare repeat content between our assembly and that of JaponicusDB, we ran a repeat masking pipeline with RepeatModeler v2.0.3 (Flynn et al., 2020) and RepeatMasker (v4.1.2-p1) (Smit et al., 2015; Tarailo-Graovac & Chen, 2009) and summarised the results using the ParseRM.pl script from the Parsing-RepeatMasker-Outputs tool (https://github.com/4ureliek/Parsing-RepeatMasker-Outputs).

### 2.5 Genome annotation

We annotated coding and noncoding genes as follows. Liftoff (v1.6.3) (Shumate & Salzberg, 2020) was used to lift over the NCBI *S. japonicus* annotation (GCF_000149845.2_SJ5) to our new assembly, allowing extra gene copies with a CDS sequence identity of 95% and over to be annotated. Using the output from RepeatMasker (see above), the generated GFF file was divided into several repeat-specific categories, namely short, A-rich, GA-rich, and LTRs (including ab initio predictions). Additionally, we calculated the concentration of these repeats in defined regions of the assembly (e.g., centromeres, telomeres, unplaced contigs, etc).

tRNAscan-SE (v2.0.12) (Chan et al., 2021) was used to identify tRNAs de novo, and barrnap (v0.9) (Seeman, 2018) and rnammer (v1.2) (Lagesen et al., 2007) were used to identify rDNA genes de novo.

To identify small nuclear RNA (snRNA) and small nucleolar RNA (snoRNA) genes, we extracted the co-ordinates for all genes of each type from the NCBI *S. japonicus* annotation, and for each type used BEDTools getfasta (v2.30.0) (Quinlan, 2014) to extract the sequences. After filtering out sequences greater than 300 nucleotides in length, we used MUSCLE (v3.8.31) (Edgar, 2004) to align the sequences and created a consensus sequence from the alignment using Mega11 (Tamura et al., 2021). Using RNAalifold (Bernhart et al., 2008) we input the consensus sequence to generate a consensus secondary structure for the alignment. Next, we used Infernal (v 1.1.4) (Nawrocki & Eddy, 2013) to reformat the aligned sequences into Stockholm format and manually added the consensus secondary structure as the metadata entry for “#=GC SS_cons.” Using the Stockholm formatted alignment file, we used Infernal cmbuild and cmcalibrate to build and calibrate our snRNA and snoRNA models and used each model to search our assembly for snRNAs and snoRNAs de novo, reformatting the output into GFF format. We repeated this pipeline using snRNAs and snoRNAs from the *S. pombe* genome and using “BEDTools intersect,” merged the output from the pipeline with that from the *S. japonicus* output, to produce unique nonoverlapping snRNA and snoRNA loci. The generated annotation files from all these tools can be found in the supplementary data (Supplementary data S1).

### 2.6 Comparison to previous *S. japonicus* assemblies

#### 2.6.1 Misassemblies

We used REAPR (v1.0.18) (Hunt et al., 2013) to map paired-end Illumina reads from *S. japonicus* (SRR16290165) to assess assembly errors across both our assembly and that of the JaponicusDB assembly. Using the Smalt aligner, REAPR maps each set of paired-end reads independently to a reference sequence and then breaks assemblies over regions it considers as being misassembled.

#### 2.6.2 Whole genome alignments

We used nucmer (Delcher et al., 2003) to align the previously allocated syntenic chromosomes to those in JaponicusDB and examined large breaks in the assembly to identify regions where our assembly had resolved more complex regions of the genome than JaponicusDB. Particular attention was paid to telomeres, centromeres, and other large areas of sequence not present in the JaponicusDB assembly.

#### 2.6.3 Telomeres

To calculate the amount of extra telomeric sequence generated, we used “delta-filter” from the MUMmer tool-suite to filter the nucmer alignments for the longest consistent set of alignments and then “show-coords” to provide a human readable version. We then took the alignments, reformatted them into BED format and used BEDTools merge to merge nonoverlapping alignments that were within 100 bps of each other. Then, using the aligned coordinates, we calculated the theoretical 5′ and 3′ extension of the telomeres (compared to JaponicusDB), taking into account JaponicusDB sequences that were not part of the alignment. For example, if an alignment between our assembly and JaponicusDB ended at position 5000, but the JaponicusDB sequence had an extra 2 kb of unaligned sequence to the 3′ end of the alignment, we extended the alignment length outwards to position 7000 and calculated the length of unaligned sequence projecting beyond the 3′ tip of the JaponicusDB telomere. We also performed this for the 5′ end of the alignments, extending the alignments to position 1 of the JaponicusDB sequence and calculating the length of unaligned sequence projecting beyond the 5′ tip of the JaponicusDB telomere.

### 2.6.4 Centromeres

The inferred centromeres in JaponicusDB were linked by stretches of N’s, 120 kb in length. To identify the amount of additional centromeric sequence in our assembly, we identified the closest protein-coding genes (with an allocated gene standard name) flanking the centromeres in JaponicusDB, calculated the amount of genomic sequence (including N’s) between the end and start of the two genes and then compared that to the distance between the same genes in our assembly. Note that here we use the term “centromere” to describe a region between the chromosome arms that is sparse in, or devoid of, protein-coding genes (and often rich in repeat content).

### 2.6.5 Coverage

For regions of interest (e.g., centromeres, rDNA arrays, large insertions, etc), we calculated long-read coverage across the regions using the following pipeline. We used minimap2 to map the ONT reads to our assembly using the pre-set parameters for mapping ONT genomic reads, and then Samtools “view” (Danecek et al., 2021) to remove alignments marked as secondary. Next, we used Samtools “depth” to count per-base coverage across all chromosomes, coding-regions of each chromosome arm (defined by the start of the first and end of the last BUSCO single-copy ortholog hit for each chromosome arm), and that of any other given interval of interest and then calculated the mean per-base coverage and standard deviation (SD) across the interval.

## 3 Results

### 3.1 A telomere-to-telomere *S. japonicus* genome assembly

**Figure 1:**
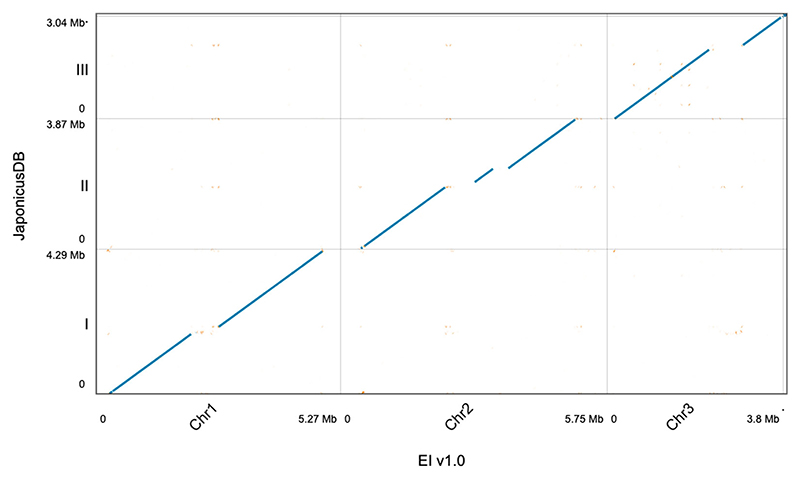
Alignment of the JaponicusDB assembly (y-axis) to EI 1.0 (x-axis). To align JaponicusDB assembly with EI 1.0, JaponicusDB was broken at the centromeres, with an additional break on Chr2. The JaponicusDB assembly has been scaffolded with N’s to join supercontigs across these areas.

Using ONT reads >50 kb, we obtained an assembly that contained three chromosome-sized contigs and one circular contig representing the mitochondrial genome. In total, the assembly comprises of 17 contigs, with an assembly size of 16,601,825 bases (after Pilon polishing) and a contig N50 of 5.27 Mb. Then, we aligned our assembly to the JaponicusDB assembly and identified three contigs that each spanned the whole length of one JaponicusDB chromosome and the mitochondrial genome. On this basis we renamed each contig in the assembly to “Chr1”, “Chr2”, “Chr3”, and “Mt” (Figure 1). Finally, we took the mitochondrial genome and trimmed off redundant overlapping ends. We refer to this final assembly as *S. japonicus* “EI 1.0.”

### 3.2 Distribution of k-mers shows low level of error

**Figure 2:**
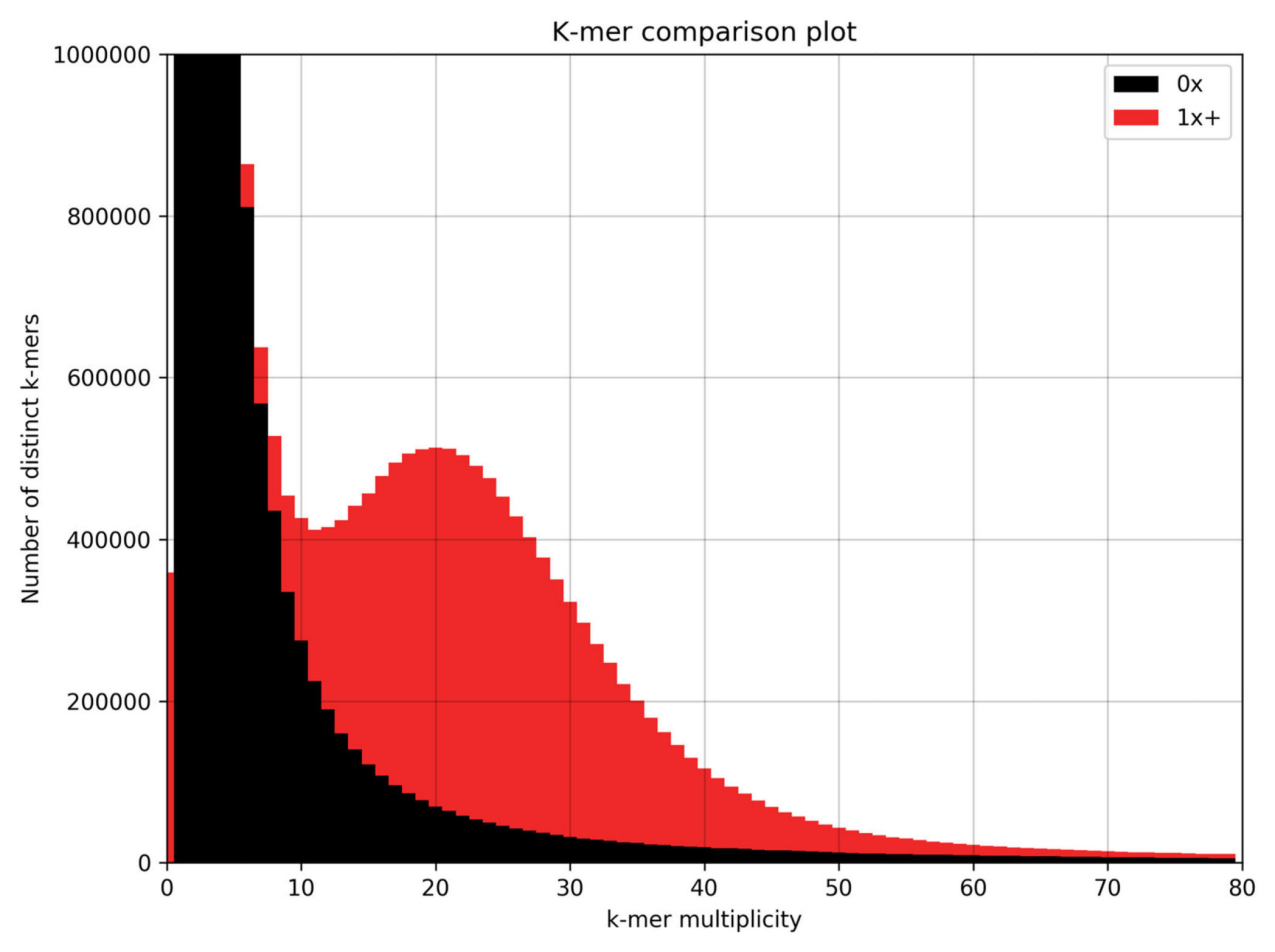
Stacked histogram of Illumina short-read k-mers present at least once (red) and not present (black) in the EI 1.0 *Schizosaccharomyces japonicus* assembly. The black peak to the left of the figure represents k-mers in the Illumina reads present in small numbers that have not been incorporated into the final assembly, and probably represent read sequencing errors. The red bar at position zero refers to k-mers found in the assembly but not in the Illumina reads, which is typically due to minor sequencing errors in the Nanopore reads incorporated into the assembly and not corrected by polishing.

We compared the k-mers in *S. japonicus* Illumina short-reads to k-mers present in the EI 1.0 assembly. The distribution of k-mers in the reads found only once in the assembly shows a normal distribution across the graph (Figure 2 in red). As is usual, there are high counts of low-copy k-mers present in the reads but not in the assembly (the black distribution to the left of Figure 2). These k-mers are likely sequencing errors in the Illumina reads that are not reflected in the EI 1.0 assembly. The black distribution of these k-mers extends along the x-axis of the graph. This represents ever-decreasing k-mers present in the reads but not present in the assembly and likely represents a small number of errors in the ONT reads not corrected by Pilon polishing.

### 3.3 Composition and chromosome association of unplaced contigs

In addition to the three chromosomes and mitochondrial genome, we retained 13 unplaced contigs that were not marked as assembly bubbles. These additional contigs ranged between 71.3 kb and 240.8 kb in length and had a combined length of 1.7 Mb (Table 1).

tig00000005 has protein coding genes SJAG_05105, SJAG_05106, and SJAG_06608. SJAG_05105 and SJAG_05106 are both present in one copy and located in JaponicusDB on the unplaced supercont5.6. SJAG_05105 is a member of the telomere-linked RecQ helicase (tlh) gene family. *S. pombe* contains four tlh genes that are located adjacent to the four telomeres of chromosomes I and II (Oizumi et al., 2021). Additionally, this contig also contains telomeric GTCTTA repeats at the 5′ end. Therefore, this contig might be an unplaced telomeric sequence from Chr 1 or Chr 2.

tig00000004 and tig00000029 have one and three copies of protein coding gene SJAG_06608 respectively, which is also present in 19 copies across the three chromosomes, and one copy on tig00000005. These two contigs also contain large tRNA arrays.

tig00000022 contains coding genes SJAG_04834, SJAG_04835, and SJAG_04836 at the far 3′ end, along with an 18S–5.8S–28S rDNA array stretching from the start of the 5′ end towards the coding genes. These three coding genes, along with the same pattern of rDNAs can be found at the end of the short-arm of Chr 3 on both EI 1.0 and JaponicusDB, so this contig appears to be a reverse complement assembly duplication from the short-arm of chromosome 3.

tig00000019, tig00000020, tig00000021, tig00000023, tig00000024, tig00000025, tig00000026, and tig00000027 all contain 18S–28S rDNA arrays across the whole length and are probably all part of the telomeric rDNA array from the short-arm of chromosome 3.

tig00000016 contains no genes or rDNA arrays but is GTCTTA-rich across the whole contig. This sequence matches the previously reported telomeric repeat for *S. japonicus* suggesting that this contig might be unplaced telomeric repeat sequences (Rhind et al., 2011).

### 3.4 EI 1.0 shows a similar resolution of single-copy orthologs to JaponicusDB

We ran BUSCO to examine the number of single-copy ortho logs recovered from both EI 1.0 and JaponicusDB. The results are quite similar, with a small increase (0.7%) of complete and single-copy orthologs recovered from EI 1.0 compared to that of the JaponicusDB assembly (Table 2). This suggests that both assemblies are equally complete in their gene content, and that very little of the assembly content is present in two or more copies.

### 3.5 Resolution of repeat content accounts for much of assembly size increase

We ran a repeat masking pipeline using RepeatModeler and RepeatMasker and summarised the results (Tables 3 and 4). We compared the repeat content of JaponicusDB to EI 1.0 (Table 3). EI 1.0 has almost five times more resolved repeat content, comprising almost 15% of the genomic content of which 11.56% are attributable to LTRs (predominantly Gypsy-type). By far the biggest difference between the two assemblies is the Helitron-like transposons that are 29 times more numerous in the EI 1.0 assembly, followed by LTRs, being 5.2 times more numerous.

The pipeline identified 9782 different repeats (where the repeat consisted of at least two bases), of which short repeats were the most numerous (4479). 482 of the short repeats were present in a single tandem array. The top three repeats present in the greatest number of copies (“CTAAGA,” “TTAGTC,” and “GTCTTA”) all represent 797 repeat copies varying around one repeat (and its reverse complement)—GACTAA, previously reported as the *S. japonicus* telomeric repeat (Rhind et al., 2011).

Additionally, we calculated the span of repeats in defined regions of the assembly (Table 5). Across the whole genome, 34.28% of the genome was identified as a repeat by RepeatMasker. Unplaced contigs and centromere (and centromere-like) regions showed a high coverage of repeat content, close to 100%. Next, telomeres (defined as the region outside the outermost BUSCO orthologs at the end of each chromosome arm), had a repeat content of approximately 71%, and the non-telomeric/centromeric part of the assembly (containing most of the protein-coding genes) had only 1.4% repeat content.

### 3.6 Enhanced tRNA gene resolution in EI 1.0

We used Liftoff to lift over the NCBI *S. japonicus* annotation (GCF_000149845.2_SJ5) to EI 1.0. The NCBI annotation contained 5215 features classified as “genes,” of which 5126 were identified in EI 1.0. Of these 5126 NCBI genes, 4980 were present in only one copy, with 146 of them present in two-copies or more, representing 2897 gene copies. Including all single and multicopy genes, a total of 7877 NCBI genes were annotated in EI 1.0 (Table 6). tRNAs represented the majority of multicopy genes. Of the 146 multicopy genes, only 19 of them were protein-coding genes (Supporting Information: Data S2), 118 of them were tRNAs, and 9 were rDNAs. Of the 19 protein-coding genes, 13 of them (representing 73 gene copies) were only partially annotated (lacking 3′ and/or 5′ UTRs). From the 89 genes that were not identified in our assembly, 81 of them were tRNAs, six of them were rDNAs, and two were partially annotated coding genes—SJAG_06621 (Tdh1) and SJAG_06597 (Tih1) (Supporting Information: Data S3).

tRNAscan-SE identified 3885 tRNAs de novo which overlapped all the 2746 annotated by Liftoff. Of these, 298 were found on Chr1, 2400 on Chr2, 408 on Chr3, 12 on the Mitochondrial genome, and 767 across three of the 13 unplaced contigs.

### 3.7 Other annotation

barrnap and rnammer were used to identify rDNA genes de novo. barrnap identified 624 rDNAs, which after merging overlapping loci accounted for 499 nonoverlapping loci. rnammer identified 494 rDNAs, all of which overlapped the 499 nonoverlapping barrnap loci. From the assembled chromosomes 250 5S rDNA loci were identified on Chr2, 12 18s–28S rDNA loci were identified on Chr3, and no rDNAs were identified on Chr1. Additionally, 237 18S–28S loci were found across 9 of the 13 unplaced contigs.

We used Infernal (v1.1.4) to annotate snRNA and snoRNA genes using previously annotated loci from both *S. japonicus* and *S. pombe*. From this, we identified 21 snoRNA loci and 11 snRNA loci, which were distributed evenly across Chromosomes 1 and 2, but only 1 snRNA locus and 2 snoRNA loci were located on Chromosome 3.

### 3.8 Comparison to JaponicusDB assembly

**Figure 3:**
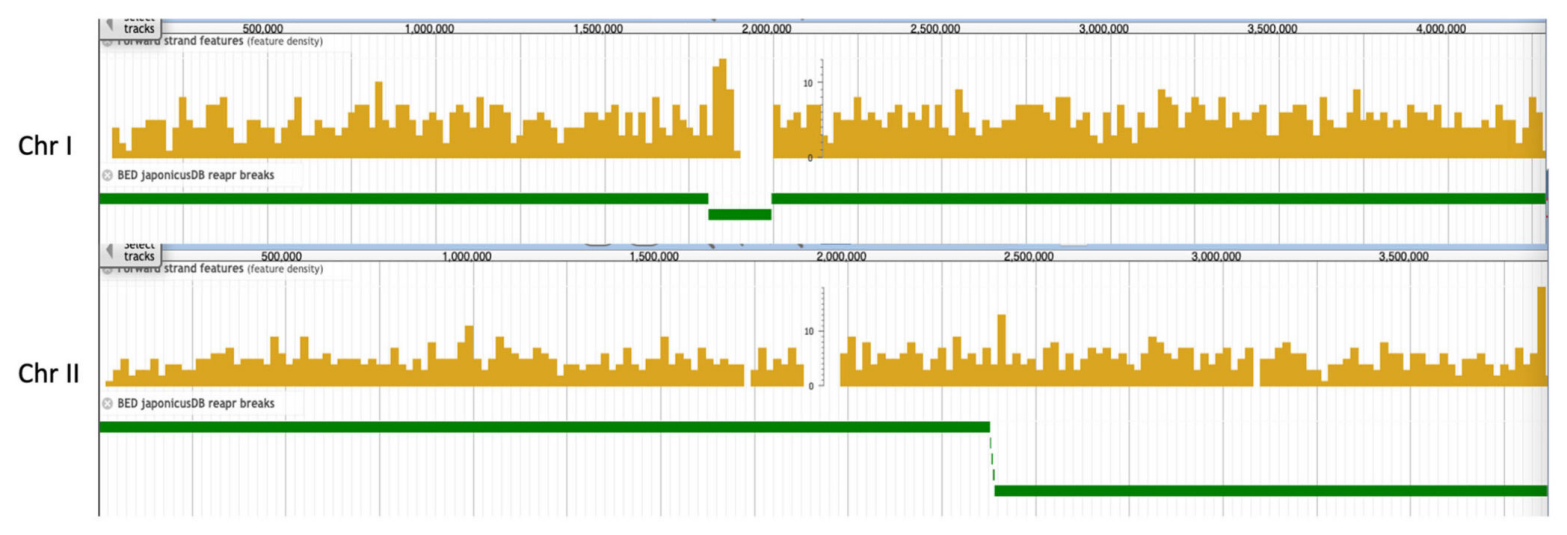
Breaks in the JaponicusDB assembly as suggested by REAPR analyses for chromosomes I and II. The yellow track is the coverage of genome features in JaponicusDB (coding genes, small RNAs, etc) and the green track represents the contigs produced in the REAPR “broken” assembly. No breaks were identified in Chromosome III.

We used REAPR to map Illumina paired-end reads to both EI 1.0 and JaponicusDB assemblies and looked for assembly errors. REAPR did not produce any breaks across EI 1.0 but produced a total of 10 breaks into the JaponicusDB assembly. Four of these breaks were in small (<200 kb) unplaced scaffolds (supercont5.4 and supercont5.5), two were in Chr I, and four were in Chr II (Figure 3). The breaks on Chr I are across the centromere and a tRNA and retro-transposon-rich flanking region, and the four breaks on Chr II were around a 30 kb region containing an array of tRNAs and rDNAs.

Considering only the assembled chromosomes, EI 1.0 totalled 16.6 Mb in length, with JaponicusDB chromosomes totalling 11.27 Mb. Using the nucmer alignment (see above), we identified differences in telomere and centromere lengths, along with other large features absent from the JaponicusDB assembly (Figure 1).

### 3.9 EI 1.0 contains 1.8 Mb of extra telomeric sequence

In total, we resolved 1.84 Mb of extra sequence at the telomeres of all three chromosomes, of which over 1 Mb was attributable to Chromosome 2 (Table 7). These regions contain a mix of telomeric and sub-telomeric sequences, with Chromosome 1 containing telomeric repeats at both ends (spanning 164 kb at the 5′ end and 370 kb at the 3′ end), and Chromosomes 2 and 3 containing telomeric repeats at their 5′ end (spanning 340 kb and 60 kb respectively).

We identified the following features of subtelomeres for each chromosome:

Chromosome 1. The 5′ (long arm) telomere was abundant in A-rich repeats, in particularly AACCCT repeats, while the 3′ (short-arm) was abundant in G-rich repeats, in particularly GTTTAGG repeats. Reverse complementing these repeats resolves both arms as containing (A)AACCCT repeats.

Chromosome 2. The final 700 kb of the 3′ (long-arm) is rich in LTRs and both telomeres are rich in short-repeats. Also, the final 575 kb is rich in tRNAs. The tRNA arrays generally followed an order of Val-Trp-Thr-Pro-Pro-His-Val-Arg-Ile-His-Ser-Cys-Leu-Pro-Lys, although a shorter version of this (with Val-Arg-Ile-His missing) was also found towards the end of the telomere.

Chromosome 3. The final 45 kb of the 3′ (short-arm) has an array of six 18S–28S rDNA repeat (but see “Coverage” later). Each repeat contains approximately 5.9 kb of rDNA genes plus a gap of 2.6 kb between genes. The 5′ (long-arm) starts with an A-rich repeat region of around 60 kb.

### 3.10 EI 1.0 contains 1.83 Mb of extra centromeric sequence

We identified protein-coding genes with gene standard names, flanking the centromeres in JaponicusDB and then compared the distance between those genes to that in EI 1.0 (Table 8). In total, we resolved 1.46 Mb of additional centromeric space than JaponicusDB. Additionally, these centromeric regions in JaponicusDB contained 373,842 Ns, therefore, the total increase in resolved chromosomal sequence is 1.83 Mb.

**Figure 4:**
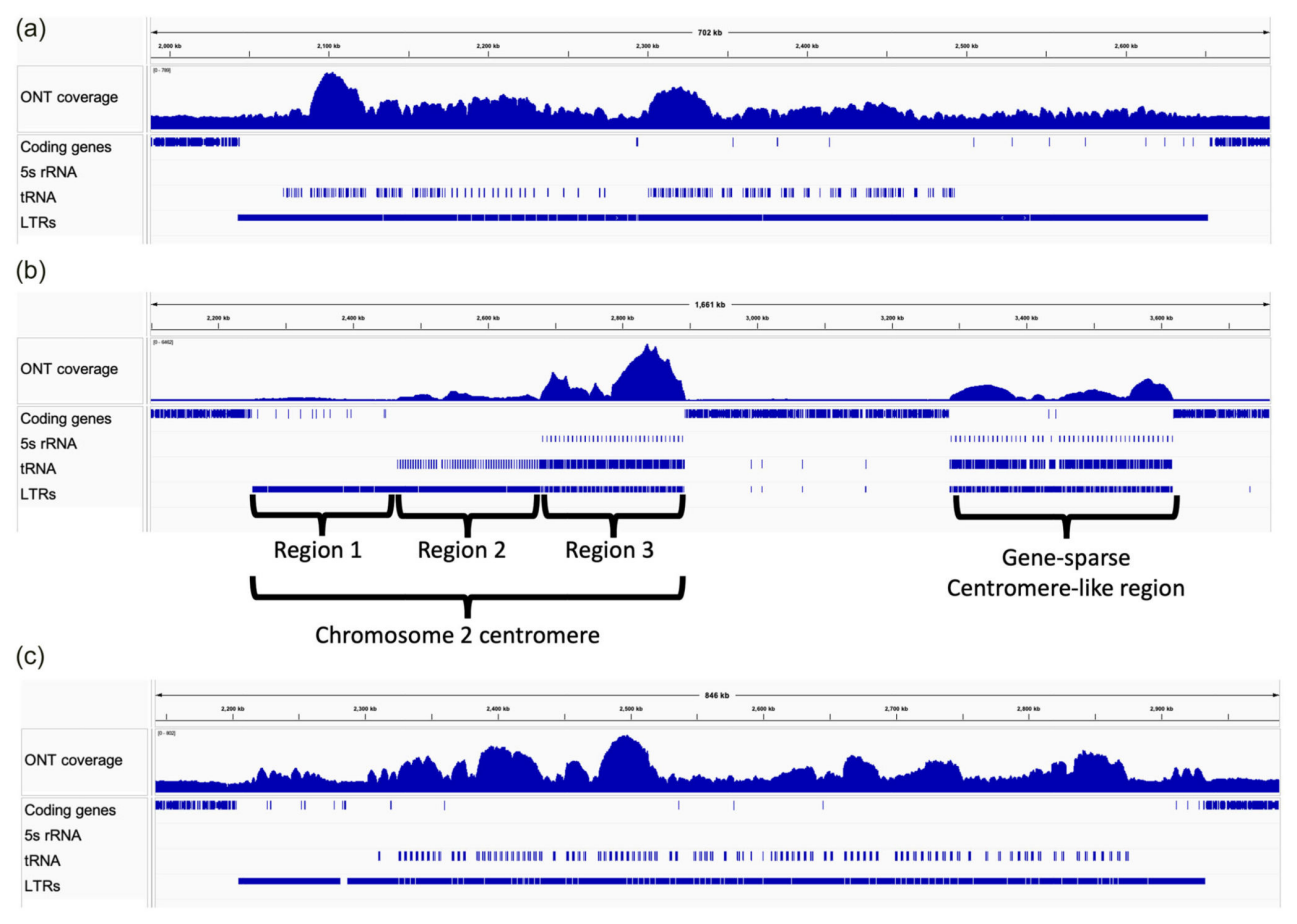
(a) ONT long-read coverage (top panel) and gene annotation over centromeres of (a) chromosome 1 (maximum read coverage 789), (b) chromosome 2 (and gene-sparce centromere-like region (maximum read coverage 6462)), and (c) chromosome 3 (maximum read coverage 802), showing bordering protein-coding genes, and de novo predicted 5S rDNA, tRNA, and LTR loci. In (b) Regions 1, 2, and 3 refer to the three regions defined in the text.

Using our annotation of genomic features, we can see that the centromere of chromosome 2 can be subdivided into three different regions (Figure 4).

Region 1: This region of around 215 kb consists of only LTRs, combined with no other repeats or rDNA genes. These LTRs continue across the whole centromere, albeit combined with other centromere-like features.

Region 2: This region spanning 211 kb, consists of repeated units of approximately 1.2 kb. They contain an Ala-tRNA, a two-exon iMet-tRNA pseudogene, and a Gly-tRNA, followed by two A-rich repeats. Each structure is separated by a 2.6 kb gap containing no tRNAs or short repeats. This region contains LTRs that are also found in Region 1 and Region 3 but contains no 5S rDNAs.

Region 3:- This part of the centromere (3′), spanning approximately 216 kb consists of 34 copies of a 5.6 kb repeated structure, each containing a T-rich and an A-rich repeat, nine tRNAs (Tyr-Leu-Glu-Phe-Arg-Lys-Gly-Ile-Gln), an array of three 5S rDNAs, followed by another array of nine tRNAs (Glu-Leu-Asn-Met-Thr-Asp-Ser-Val-Ala) (Figure 4).

tRNA arrays on Chromosome 3 differed from Chromosome 2 by being much shorter, occurring in an array of five re-occurring tRNAs (Gln-Pro-Thr-Ser-iMet(pseudogene)-Gln). tRNA arrays on Chromosome 1 tended to occur in 3 kb repeated tRNA structures (Ala-iMetAla-Arg-Pro-Gly), although these structures tended to be reversed at the 3′ end of the centromere.

### 3.11 Chromosome 2 contains a centromere-like gene sparse region

**Figure 5:**
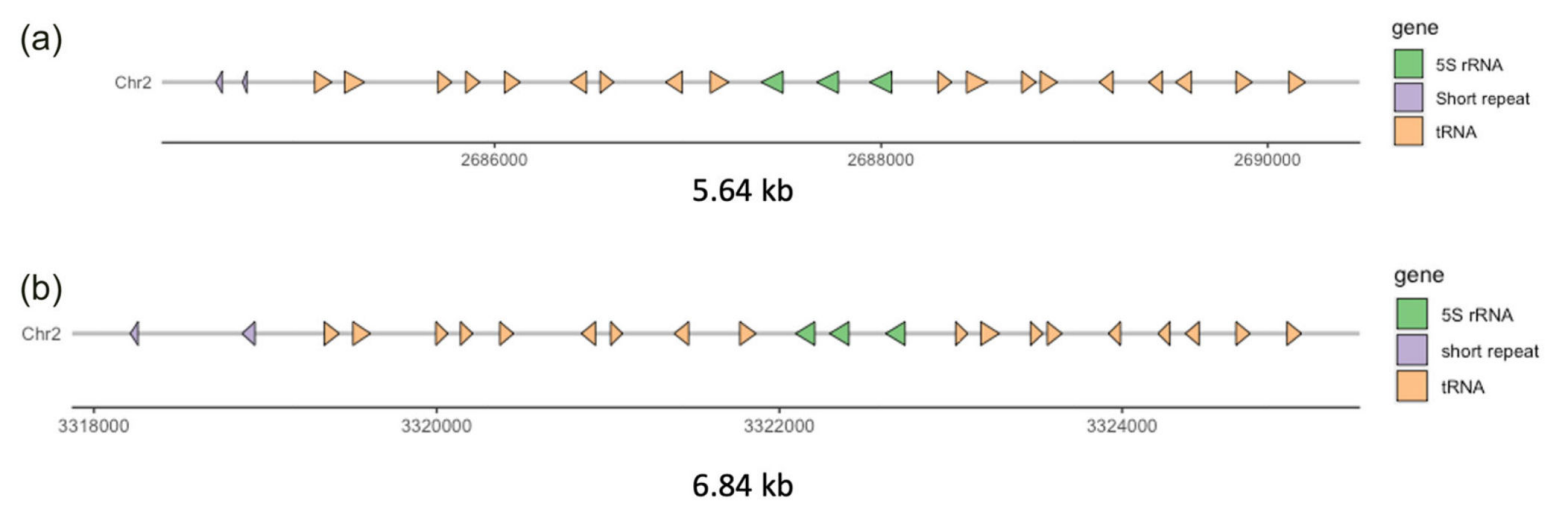
Typical structure of a tRNA array from Chromosome 2 in (a) the centromere region 3, and (b) the centromere-like gene sparse region (LTRs not shown). The array starts with two short repeats, followed by nine tRNAs, three 5S rDNAs, and ends with nine more tRNAs.

We located a region of approximately 331 kb on Chromosome 2 that had not been resolved in previous *S. japonicus* assemblies. This region is rich in both tRNAs and 5S rDNAs and only contains one protein-coding gene (in two copies) - SJAG_06608, annotated as a chromo/chromo shadow domain family gene. This part of the chromosome consisting of 46 repeated 6.4 kb tRNA/rDNA structures almost identical to that found at the 3′ end of the centromere (Region 3 above) and differed only in that they tended to be longer (e.g., 6.84 kb, compared to 5.64 in the centromere), and the two short repeats were both T-rich and were further apart than in the centromere (Figure 5).

### 3.12 Coverage analyses suggests collapsed tRNA and 5S rDNA arrays

We calculated long-read coverage across all three chromosomes along with that for only the coding region (Table 9) and compared it to that of other regions of interest on chromosome 2 (Figure 4 and Table 10).

Mean long-read coverage across the three chromosomes varied between 161x to 172x, with standard deviations (SD) of between 40 and 94. When only considering coding regions, the coverage was much more even. Mean coverage for coding regions varied between 173x to 176x, with SD of between 15 and 17. The mean coverage across only the coding regions of Chromosome 2 was 176 (SD 17), while that of the centromere was 248 (SD 240), suggesting large variation in coverage between coding and noncoding regions. We split the centromere into three regions, paying particular attention to Regions 2 and 3 (Table 10). Region 2 had coverage slightly below both the centromere mean and whole chromosome mean, and Region 3, which also contained tRNAs and a large array of rDNAs, had coverage 2.7 times that of the chromosome mean (Table 10 and Figure 4). We also examined coverage over the 331 kb centromere-like gene sparse region, which had coverage slightly above the mean, albeit with a high SD. Taking coverage into consideration, Region 2 might have a slightly shorter length of 135 kb and Region 3 might have a greater length of 601 kb, increasing the estimated centromere size from 642 kb to 952 kb.

### 3.13 Chromosome 3 telomeric rDNA array is likely not fully resolved

Additionally, we also looked at 18S–28S rDNA arrays in the assembly. These were present in a 49 kb region at the 5′ end of Chromosome 3 and the 71 kb unplaced contig tig00000022. tig00000022 is a reverse complement assembly duplication of the 3′ end of Chromosome 3 and together likely represent a collapsed 18S–28S rDNA array that was not merged during assembly. Additionally, eight other unplaced contigs contained only 18S–28S rDNA arrays (Table 11). The cumulative length of the assembled 18S–28S rDNAs is 1.05 Mb. Assuming that all these sequences represent a single telomeric 18S–28S rDNA array from Chromosome 3 and accounting for coverage and length of these sequences, it is likely that the actual size of this rDNA array is approximately 2.25 Mb in length (Supporting Information: Table S4).

## 4 Discussion

### 4.1 Genome size

We have sequenced, assembled, and annotated a de novo genome assembly for *Schizosaccharomyces japonicus* (EI 1.0), which is more complete and contiguous than previous versions. Each chromosome is represented by a single telomere-to-telomere contig. The chromosomes (totalling 14.8 Mb) contain roughly 3.6 Mb (30.5%) more sequence than the chromosomes in the current reference sequence (JaponicusDB SJ5, size 11.2 Mb). This additional sequence consists of 1.84 Mb more sub-telomeric sequence, 1.45 Mb more centromeric sequence and a 331 kb centromere-like region on chromosome 2 that had not been previously described. The assembly has 1.97 Mb more repeat content than JaponicusDB, therefore >50% of the size increase is down to better resolution of repeat content or repeat-rich regions (i.e., repeats, telomeres, and centromeres), rather than gene content (EI 1.0 contains only 0.7% more single-copy orthologs than JaponicusDB). When accounting for coverage over centromeres and telomeres, the true genome size might be closer to 18.12 Mb (Supporting Information: Table S4).

### 4.2 Chromosome 2 centromere-like gene sparse region

The three assembled chromosomes in the JaponicusDB assembly were inferred from genetic linkage within the seven largest super-contigs, with the inferred centromeres linked by stretches of 120 kb of N’s. The centromere gaps can be seen in the genome alignments for all three chromosomes (Figure 1). The final two supercontigs to be closed on the JaponicusDB assembly were on chromosome 2 (supercontigs 7A and 5D), which corresponds to the 331 kb gene-sparse region we identify on chromosome 2. This is approximately 390 kb downstream from the centromere and both features are shown as gaps in the genome alignments (Rhind et al., 2011; Supporting Information S1: Figure 2, and Figure 1). Further evidence of this region not being properly assembled in JaponicusDB is shown by the REAPR analysis which shows four misassembly breaks across this region (Figure 3). This region displays most resemblance to the 3’ end of the centromere on the same chromosome, characterised by being rich in both 5S rDNAs and tRNAs across its full length, of which the tRNA array order is identical to that of the centromere. However, the composition of and the distance between the short repeats differs from that of the centromere, providing confidence that this region is distinct and correctly assembled. The centromeres of chromosomes 1 and 3 are rich in tRNAs and LTRs but have no rDNA arrays, suggesting that the centromere-like gene sparse region on chromosome 2 originated from the centromere of chromosome 2. Long-read coverage analyses of the region suggests that the actual length may be 1.3x longer, around 430 kb. Liftoff annotated 43 copies of the rDNA gene SJAG_16156 across this region. SJAG_16156 can be located on JaponicusDB Chromosome II and is part of a 13 kb rDNA/tRNA array (containing 3 rDNAs and 7 tRNAs) flanked by genes SJAG_04837 and SJAG_04436. These two genes also flank the 331 kb centromere-like gene sparse region, providing evidence of the collapsing of rDNA and tRNA arrays in JaponicusDB.

### 4.3 tRNA arrays

Long arrays of tRNAs are found across the majority of each centromere and the centromere-like gene sparse region on Chromosome 2. The exceptions to this are in the centromere of Chromosome 1, where they are absent from the last 160 kb, and the centromere of Chromosome 2, where they are absent from the first 215 kb (Figure 4). There is also a 575 kb tRNA array on the 3′ telomere of Chromosome 2. Additionally, tRNAs are also present on unplaced contigs tig00000004, tig00000005, and tig00000029. Compared to previous assemblies, EI 1.0 has 2623 extra annotated copies of tRNA genes, comprising 90% of all novel annotated genes in this assembly. It had been proposed that *S. japonicus* contained no tRNAs with Ala (AGC) anticodon, which is the most abundant in other yeasts, and decoded the GCU Ala codon exclusively through a species-specific wobble Ala(TGC), and Ala(CGC) (Iben & Maraia, 2012). We indeed found Ala(TGC) (106 copies across all three chromosomes) and Ala (CGC) tRNAs (47 copies, all on Chromosome 1). However, we also discovered 80 copies of Ala(AGC), all situated on Chromosome 2, within the centromere and the centromere-like gene sparse region. Both of these regions are poorly resolved in the previous JaponicusDB SJ5 assembly, providing further evidence of the advantages of long-read sequencing assembly. Additionally, tRNAscan-SE identified 12 tRNAs on the mitochondrial genome (along with five tRNA pseudogenes). This is notably less than the 25 reported by a previous study (Bullerwell, 2003).

### 4.4 rDNA arrays

EI 1.0 contains three regions containing repeated copies of rDNA arrays—two 5S rDNA arrays on Chromosome 2 (one in the centromere, and one in the centromere-like gene sparse region), and one 18S–28S rDNA array on the 3′ (short-arm) of Chromosome 3. rDNAs are absent from Chromosome 1. The 5S rDNAs in the centromere of Chromosome 2 are restricted to a 216 kb tandem array at the 3′ end, which is represented by 34 repeated modules of three 5S rDNAs (with one exception, where only one 5S rDNA is present). The centromere-like gene sparse region on Chromosome 2 has a 5S rDNA tandem array made up of 46 repeated modules of between one and four rDNAs in each module. Modules with three rDNAs dominated the first 116 kb of this region, with modules of four rDNAs dominating the final 215 kb. In all but three modules, the first rDNA shows a high degree of similarity to JaponicusDB gene SJAG_16156. In each of the three exceptions, the rDNA module contained only one or two rDNAs. The remaining rDNAs in each module did not have homologous genes in JaponicusDB (defined by the lack of Liftoff annotations over each one), suggesting a lack of rDNA array resolution in that assembly. Chromosome 3 has a 45 kb array of 18S–28S rDNAs at the very end of the short-arm telomere. The array is formed of six modules of reoccurring 18S–28S pairs. This is the only place in the assembled chromosomes that has this pattern of rDNAs, but it should be noted that 9 of the 13 unplaced contigs also have the same pattern of 18S–28S pairs across their full length (including tig00000022, which is a reverse complement of the 3′ end of chromosome 3). The cumulative length of these contigs is approximately 1 Mb (2.25 Mb when adjusted for coverage) and likely represent unscaffolded regions of the Chromosome 3 sub-telomere. This is consistent with the physically marked position of the 18S–28S rDNA array in *S. japonicus* (Yam et al., 2013).

We have completed the first telomere-to-telomere genome assembly of the fission yeast *S. japonicus* and described a hitherto undescribed genome architecture. Previously unseen features include vastly expanded repeat arrays, including those encoding tRNAs and rDNAs, compared to other fission yeast species. Another notable feature is the centromere-like gene-sparse region on the long arm of chromosome 2, with as yet unknown implications for the evolution of centromeres and perhaps chromosome number changes. It will be interesting to explore the mechanisms and evolutionary pressures that have led to and fixed this huge increase in repeat number, as well as how they contribute to the physiology and life-style of this unique model species.

This telomere-to-telomere genome assembly required the resolving of complex repetitive features (including tRNA and rDNA arrays) which was possible due to the availability of long ONT sequencing reads (>50 kb). However, even with such long reads, coverage analysis demonstrates and quantifies some collapse of the highly repetitive regions (Figure 4, Tables 10, and 11). It is worth noting that there is likely to be diversity in repeat copy number between *S. japonicus* isolates and even within a culture. Understanding these differences will be aided by increases in nanopore sequencing read length and accuracy. Nanopore sequencing generates read lengths that reflect the length of purified DNA fragments with (theoretically) no limit on read length. Therefore, in the future, nanopore sequencing datasets with even longer reads will allow increasingly accurate genome assemblies and shed more light on as-yet undiscovered genomic diversity.

## Supplementary Material

Supporting Information

Additional supporting information can be found online in the Supporting Information section at the end of this article.

Supplementary data S1

Supplementary data S2

Supplementary data S3

Supplementary data S5

Supplementary table S4

## Figures and Tables

**Table 1:** Contig-specific lengths and GC-content for each sequence in the *Schizosaccharomyces japonicus* EI 1.0 assembly.

Contig name	Length (bps)	GC content
Chr1	5,271,478	0.439
Chr2	5,749,449	0.435
Chr3	3,796,598	0.447
Mt	80,043	0.198
tig00000004	180,911	0.450
tig00000005	240,835	0.422
tig00000016	138,683	0.404
tig00000019	156,967	0.422
tig00000020	174,897	0.423
tig00000021	108,239	0.422
tig00000022	71,327	0.416
tig00000023	141,633	0.423
tig00000024	78,416	0.418
tig00000025	86,660	0.422
tig00000026	84,135	0.423
tig00000027	97,271	0.423
tig00000029	144,283	0.460

*Note*: The mean GC content of the 16 non-mitochondrial contigs was 0.428 and 0.44 across the three chromosomes.

**Table 2:** Comparison of the number of single-copy orthologs identified in EI 1.0 and JaponicusDB.

BUSCO category	EI 1.0 (%)	JaponicusDB (%)
Complete	1360 (79.7)	1349 (79.0)
Complete and single-copy	1308 (76.7)	1297 (76.0)
Complete and duplicated	52 (3.0)	52 (3.0)
Fragmented	45 (2.6)	49 (2.9)
Missing	301 (17.7)	308 (18.1)
Total	1706	1706

*Note*: “Complete and duplicated” orthologs are an indication of duplication in the assembly (e.g., through incorrectly assembling a region of the genome two times or more), “fragmented” refers to genes that are identified but not full-length, and “missing” is an inferred metric of those genes not found in the assembly.

**Table 3:** Comparison of assembly repeat content (number of nucleotides masked and % of the genome) by class between JaponicusDB and EI 1.0.

Repeat class	JaponicusDB	EI 1.0
SINE	20,709 (0.18%)	17,450 (0.10%)
LTR	369,855 (3.13%)	1,919,713 (11.56%)
RC (Helitron)	11,497 (0.09%)	334,938 (2.02%)
Unknown	101,426 (0.86%)	200,590 (1.21%)
**Total masked**	**503,487 (4.26%)**	**2,472,691 (14.89%)**

Abbreviations: LTR, long terminal repeat; RC, rolling circle transposons (Helitron-like transposons); SINE, Short interspersed nuclear elements.

**Table 4:** Counts of repeats identified by the RepeatModeler—RepeatMasker pipeline.

Repeat type	Count
Short	4479
LTR	4362
A-rich	600
G-rich	246
GA-rich	95

**Table 5:** Repeat content for defined regions of the EI 1.0 assembly.

Feature	Repeat bps	% repeat coverage
Whole genome	5,714,072	34.28
Chr2 centromere-like gene sparse region	330,225	99.74
Unplaced contigs	1,697,395	99.02
Centromeres	1,935,458	96.84
Telomeres	2,250,807	71.1
Non-centromeric/telomeric	10,214,646	1.4
Mitochondria	2537	3.22

*Note*: Overlapping repeats were merged, so as to not artificially inflate repeat content calculation.

**Table 6:** The number of single- and multi-copy NCBI genes identified by Liftoff in EI 1.0.

NCBI genes	All genes	Protein coding	tRNAs	rDNAs
Only one copy	4980	4857	123	0
Multicopies	146 (2897)	19 (85)	118 (2623)	9 (189)
Total copies	7877	4942	2746	189

*Note*: Multicopy genes are the number of genes that are present two times or more, with the number in brackets representing the total number of copies present.

**Table 7:** The estimated length (bps) of additional genomic sequence resolved at the telomeres of EI 1.0 when compared to JaponicusDB.

Chromosome	5’ arm extension	3’ arm extension	Total
1	274,257	339,994	614,251
2	414,184	636,974	1,051,158
3	162,411	14,807*	177,218
Totals	850,852	991,775	1,842,627

*Note*: *Denotes the highly-collapsed 18S–5.8S–28S rDNA array that might be approximately 2.25 Mb in length.

**Table 8:** The distances and differences between the innermost centromere-flanking protein-coding genes of the JaponicusDB assembly and EI 1.0.

Chr	Left-flank	Right-flank	JaponicusDB distance	EI 1.0 distance	Difference
1	spt7 (SJAG_02927)	rpm1 (SJAG_06586)	230,714	610,170	379,456
2	pptl (SJAG_06603)	rad31 (SJAG_05032)	160,850	644,818	483,968
3	ser2 (SJAG_04449)	rga7 (SJAG_01114)	145,988	738,277	592,289
Totals			537,552*	1,993,265	1,455,713

*Note*: *Includes 373,842 Ns.

**Table 9:** Mean per-base coverage and standard deviation (SD) of ONT long-reads across whole chromosomes and coding regions only of EI 1.0.

Chr	Whole chromosome (SD)	Coding regions (SD)
1	161 (40)	176 (15)
2	172 (94)	176 (17)
3	168 (78)	173 (16)

*Note*: Coding regions are defined by the start position of the first and end position of the last BUSCO ortholog on each chromosome arm.

**Table 10:** Mean per-base coverage and standard deviation (SD) of ONT long-reads and across centromere features and centromeric-like regions on chromosome 2.

Interval (length)	ONT coverage (SD)	Comment
Chr2:2250038-2891897 (642 kb)	248 (240)	Chr2 centromere
Chr2:2250038-2464999 (215 kb)	151 (16)	Region 1 - Chr 2 LTR region
Chr2: 2465000-2675532 (211 kb)	111 (16)	Region 2 - Chr 2 centromere tRNA array
Chr2:2675533-2891897 (216 kb)	479 (301)	Region 3 - Chr 2 centromere tRNA and rDNA arrays
Chr2:3285699-3617048 (331 kb)	202 (94)	Chr2 centromere-like gene sparse region

**Table 11:** Mean per-base coverage and standard deviation (SD) of ONT long-reads across 18S–28S rDNA arrays.

Interval	Mean coverage (SD)	Comment
Chr3:3749417-3796598 (47 kb)	725 (337)	Chromosome 3 telomeric 18S-28S rDNA array
Unplaced 18S-28S rDNA arrays (1 Mb)	377 (398)	9 unplaced contigs with 18S-28S rDNA arrays across most of sequence.

## Data Availability

All new data for the assembly can be located under ENA Project PRJEB63404. Individual chromosomes and the mitochondrial genome are accessions as OY101111-OY101114, and the unplaced contigs can be found under accession number CATPIX010000000.

## Take-away

- A telomere-to-telomere genome assembly of the fission yeast *Schizosaccharomyces japonicus*.
- Chromosome 2 harbours a previously unknown second centromere-like region.
- The estimated genome size of *S. japonicus* may be up to 18.12 Mb.
